# MALDI-TOF mass spectrometry biotyping: At line monitoring of recombinant CHO cell cultures

**DOI:** 10.1186/1753-6561-9-S9-P53

**Published:** 2015-12-14

**Authors:** Sebastian Schwamb, Philipp Wiedemann

**Affiliations:** 1 Center for Applied Biomedical Mass Spectrometry (ABIMAS), Mannheim, Baden-Württemberg, 68163, Germany; 2 Mannheim University of Applied Sciences, Mannheim, Baden-Württemberg, 68163, Germany; 3 current address: Sanofi-Aventis Deutschland GmbH, Biorealization Germany, Bioprocesses & Manufacturing, Frankfurt am Main, Hessen, 65926, Germany

## Background

The cultivation of mammalian cells, especially Chinese Hamster Ovary (CHO), has found widespread acceptance for the production of biopharmaceuticals. Process monitoring - enforced by the PAT initiative of the FDA - underwent major changes from examining the bioreactor/culture as a "black-box" towards integrated methods, helping to gain deeper process understanding by assessing the cells themselves.

Previously, we presented first results of CHO batch cultures assessed in order to monitor cell stress and early apoptosis by Intact Cell MALDI-TOF Mass Spectrometry (ICM MS) biotyping, a method originally used in clinical microbiology [1]. Now, we broadened its application and addressed additional CHO cell lines (DXB11 and CHOK1), different cultivation-formats (uncontrolled Erlenmeyer flasks vs. lab scale bioreactors) and operation modes (Batch vs. Perfusion). Furthermore, we transferred the method to a compact, "low-priced" mass spectrometer (MicroFlex, Bruker Daltonik, Bremen, Germany). The experimental aim was to establish characteristic MS patterns indicative for different apoptotic states with emphasis on detection of early apoptosis ubiquitously applicable independent of CHO-subline, cultivation-format, -mode or instrumental equipment (MS).

## Materials and methods

The presented data set was generated working with a suspension adapted CHO K1 cell line. Exponentially growing pre-culture was used to inoculate in parallel two bioreactors operated in different cultivation modes (batch vs. perfusion).

An exponentially growing CHO suspension cell line was inoculated at a seeding density of 4-5*105 c/ml and an initial cultivation volume of 700 ml and cultivated over in total 192 h.Exemplary results from one biological experiment of batch and perfusion cultivation in such a controlled bioreactor environment are shown.

Perfusion mode was realized byinstallinga BioSep 1L (Applikon Biotechnology B.V., Delft, Netherlands) on the head plate of the bioreactor in a 12 mm port. Between 48 h and 144 h, 1.2 working volumes medium per day were exchanged in continuous mode.

Samples for assessing viability-/apoptosis progression and for MS analysis were taken at 24, 48, 72, 96, 120, 144, 152*, 160*, 168 and 192 h (* only for cultivation in perfusion mode) during the experiments. Viable cell density and cell viability were determined by trypan blue dye exclusion using a ViCell cell counter (Beckman Coulter, Krefeld, Germany). Apoptosis was assessed by means of caspase-9 activity(Promega Caspase-Glo®9 assay kit) using a microplate format (plate reader POLARstar Omega, BMG Labtech, Ortenberg, Germany).Label-free MS apoptosis-monitoring was carried out using a Bruker Autoflex III MALDI-TOF/TOF MS. Samples were prepared from as little as 2500 cells. The method is described in detail in Schwamb et al. 2013[1].

## Results

In accordance with previously shown data and procedures [1], current batch and perfusion mode cultivations where analyzed by ICM MS and by standard analytical methods in parallel, in terms of determining the capability/sensitivity of monitoring early events of apoptosis.

**Figure 1:**
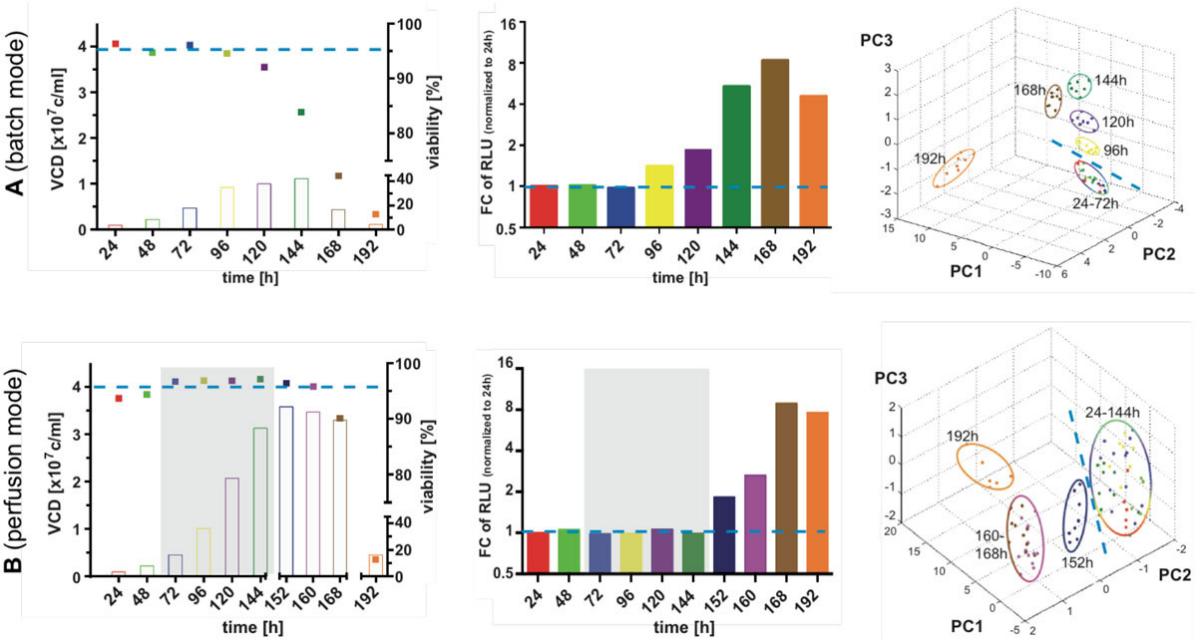
**Viable cell density and viability (1), caspase 9 activity (2) and PCA of ICM MS biotyping (3)**. Batch (a) and perfusion mode (b). VCD: viable cell density; FC RLU: fold change of relative luminescence units; PC: principal component of the respective analysis. (1) closed symbols, VCD and open symbols, viability (3) each dot represents one technical replicate (up to eight technical replicates for one biological sample). Grey rectangle in B (1) and (2) indicates the time span of continuous medium exchange. Dashed lines illustrate at which point culture alteration is detectable with respective method.

As shown in Figure 1 (1) cell viabilities as assessed by trypan blue remained constant over 96 h for batch- and 160 h for perfusion-cultures. A first drop in cell viability was noticed between 96 and 120 h for batch-(Figure 1 A (1)) respectively 160 and 168 h for perfusion-culture (Figure 1 B (1)).

In ICM MS analysis, a total of approx. 170m/z (for both Batch and Perfusion) values were monitored in a mass to charge (m/z) range of 4,000 to 30,000. Principle component analysis (PCA; Figure 1 A (3) and B (3)) of ICM MS results showed no clear group discrimination during the first 72 h of batch- and first 144 h of perfusion-cultivation. In contrast, cell samples obtained from 96 h (Batch) and 152 h (Perfusion) onwards of corresponding cultivations appear as distinct groups in PCA analysis.

Monitored Caspase 9 activity (Figure 1 A (2) and B (2)) began to increase from 72 h of batch- and 152 h of perfusion-culture on, i.e. concomitantly with PCA analysis (Figure 1 A (3) and B (3)).

In conclusion, the at-line analysis of ICM MS Biotyping (Fig. 1 A (3) and B (3)) for monitoring alterations in the MS spectra shows results concomitant with specific Caspase 9 signals indicative for the onset of early apoptosis. Furthermore, it does so independent of mode of bioreactor operation. This allows detection of cell viability changes up to 24h earlier by using ICM MS compared to standard culture monitoring method (trypan blue).

Closer data analysis proved the scale and operation mode independency of formerly identified apoptosis specific subset of m/z values. A previously developed [1] classification model, based on MS data of uncontrolled batch cultures, was successfully used toward classification (viable or apoptotic/necrotic) of current cultivations under controlled conditions (batch and perfusion). The classification power is exemplary illustrated for the perfusion culture samples as positive predictive value (PPV) which is the number of correctly classified samples over the total number of classified (Table 1). All biological samples were analyzed by ICM MS as 6-8 technical replicates, meaning in theory a PPV > 50% is sufficient for classification.

**Table 1:** Classification power of "unknown" samples(up to eight technical replicates per biological sample) from perfusion mode using the CPT model built with spectral information from samples cultivated under batch mode. #V: number of technical replicates classified as viable; #A/N: number of technical replicates classified asapoptotic/ necrotic.

"unknown" sample	Drop of viability detected by Trypan blue	Apoptosis assessment by activity assessment of Caspase 9	Result of unsupervised classification based on spectral information		Mean Positive Predictive Value (PPV)
**[h]**	**[Y/N]**	**[Y/N]**	**#V**	**#A/N**	**[%]**
**24**	N	N	6	2	78
**48**	N	N	5	1	
**72**	N	N	5	1	
**96**	N	N	6	1	
**120**	N	N	4	3	
**144**	N	N	6	1	
**152**	N	Y	/	8	100
**160**	N	Y	/	6	
**168**	Y	Y	/	7	
**192**	Y	Y	/	6	

## Summary and Conclusions

We successfully proved the scalability (Erlenmeyer flask vs. bench-top-scale bioreactor; data not shown) and operation mode independent applicability of ICM MS (Fig. 1) focussing on at-line apoptosis and cell viability determination. The method indicates onset of apoptosis as early as a sensitive, albeit not at-line capable Caspase 9 detection assay.

This adaptability, in combination with further investigations e. g. method transferability to more compact and lower-priced MS (recent, unpublished data indicate transferability to a Bruker Biotyper MS; data not shown) might be of impact for potential users in the field.

Although we focussed on apoptosis induction, in the future it might be possible to expand the method towards detection of other pattern associated cellular states. The fast, robust and automated acquisition of cell state specific MS signatures together with simple, label free sample preparation could become a promising tool for at-line CHO culture monitoring.
